# Predictive Value of Emergency Designation on Outcomes of Moribund Patients

**DOI:** 10.7759/cureus.26875

**Published:** 2022-07-15

**Authors:** Zachary A Turnbull, Virginia E Tangel, Peter A Goldstein

**Affiliations:** 1 Anesthesiology, Weill Cornell, New York City, USA

**Keywords:** discharge disposition, asa status, length of hospital stay (los), perioperative mortality, asa ps ( american society of anaesthesiology physical status)

## Abstract

Background: Anesthesiologists are increasingly encountering sicker patients that require potentially life-saving surgical interventions, and assess risk using the American Society of Anesthesiology Physical Status (ASA PS) classification system. Here, we examined long-term mortality along with hospital length of stay (LoS) and discharge disposition for survivors in ASA PS 5 and 5E patients.

Methods: Adult surgeries were extracted from New York-Presbyterian Hospital/Weill Cornell Medical Center’s Electronic Medical Record (EMR) for cases between January 1, 2013 and December 31, 2017; outcomes were collected from EMRs and the Social Security Death Index Master File.

Results: 194,947 cases were identified. Mortality correlated with increasing ASA PS; the same trend was observed within both emergent and non-emergent sub-populations. Two hundred seventy-six cases were identified as 5/5E. This patient population had a higher rate of mortality at 30 days than at 48 hours (25.9% vs. 13.4%, respectively, p < 0.01); there was no difference between survivor functions at 30 or 90 days (p = 0.63, p = 0.09, respectively). Survivors within the 5 or 5E subpopulations did not have significantly different LoSs. Further, survivors after 90 days typically had a disposition of hospice, long-term facilities, inpatient rehabilitation, or self-discharged.

Conclusions: Mortality increases with increases in ASA PS classifications. There is no difference in outcomes for 5 vs 5E at 30- or 90-day postoperatively. Similarly, emergency status did not play a role in LoS. Most 5 or 5E patients are not discharged home but to another facility. These outcomes should be considered during the informed consent process in this high-risk surgical population.

## Introduction

The increasing burden of illness in hospitalized patients is resulting in a high-acuity patient population that is ever more likely to require surgical intervention [1]. Consequently, anesthesiologists are required to navigate more challenging cases, especially those that have life-saving implications. Given that end-of-life healthcare costs are continuing to rise [2,3], it is imperative that anesthesiologists and surgeons understand and present the full range of likely outcomes of lifesaving measures when offering moribund patients and their families the option of surgical intervention.

Although there are concerns with its reliability and validity, the American Society of Anesthesiologists (ASA) Physical Status (PS) classification [4-10] has shown utility in predicting outcomes after surgery at both 48 hours [11] and 30 days postoperatively [12]. Recently, Hopkins et al. reported an association between increasing ASA PS status and 48-hour postoperative mortality for both elective and emergent procedures [11].

Within the ASA PS classification schema, ASA PS 5 denotes a “moribund patient who is not expected to survive without the operation,” and the modifier “E” denotes an emergency surgery: one in which a delay in treatment would lead to a significant increase in the threat to life or limb. In the study by Hopkins et al., patients who were classified as ASA PS 5 had a 48-hour mortality rate of ~6%, whereas those classified as ASA PS 5E had a 48-hour mortality rate of ~20% [11]. These data show an improvement in survival compared to the outcomes observed in the 1990s, with a fraction of deaths occurring more than two weeks postoperatively [13]. Interestingly, there was an increase in mortality in the 1990-1993 (0%) to 1995-1997 (15.5%) time frame, suggesting an increase in underlying disease burden within the space of ten years. Longer-term outcomes in this high-risk population of patients identified as ASA PS 5 or 5E are unknown.

We sought to expand the results of the Hopkins’ study to assess mortality beyond 48 hours. Though useful, 48-hour survival outcomes provide incomplete information to patients, families, and those consenting to lifesaving measures when making difficult and emotionally painful decisions. We also aimed to assess the overall hospital length of stay (LoS) and discharge disposition for individuals who survive beyond 48 hours in an effort to understand the broader impact of surgical intervention in a high-risk surgical population.

The primary objective of the study was to identify the difference in mortality rate of ASA PS 5 and 5E patients at 48 hours, 30 days, and 90 days following surgery using local EMRs. The secondary objectives were to identify the distribution of these patients across discharge dispositions and LoS based on emergency status.

## Materials and methods

Study database and population

We extracted patient data from New York-Presbyterian Hospital/Weill Cornell Medical Center’s electronic medical records (EMR: CompuRecord®; Philips Medical Systems, Andover, MA & Allscripts Healthcare Solutions, Inc., Chicago, IL) with IRB approval. Variables included ASA PS, elective or emergent (as assigned by the anesthesia team at the time of surgery), procedure type, and age. Records included all surgical procedures in which anesthesia was administered occurring between January 1, 2013 and December 31, 2017. Excluded were pediatric cases (under 18 years of age), ASA PS 6 cases, cases with missing ASA PS status data, duplicate cases, and cases with negative or missing values for LoS. Inpatient and outpatient hospital LoS, discharge location, and/or date of death, if applicable, were matched to these records from several sources: our inpatient EMR (Allscripts); our outpatient Electronic Health Record (Epic Systems Corporation, Madison, WI); and the United States Social Security Death Index Master File (National Technical Information Service).

Statistical analyses

Patient demographics and surgical characteristics were assessed with the Mann-Whitney U test or Fisher’s exact test, as appropriate. Surgeries were identified by CPT code and categorized as abdominal. airway, cardiac/aortic, gastrointestinal (GI) endoscopy, integumentary, interventional radiology, neurosurgery, obstetrics-gynecology, thoracic, urology, and vascular.

Following published methodology [11], incident death within 48 hours and 30 days of procedure was cross-tabulated by ASA PS classification in the full cohort, as well as separately for elective and emergent procedures. Using the ordinal properties of ASA PS, a Cochrane-Armitage trend test was used to evaluate the association between increasing ASA PS and all-cause mortality. A chi-square test was used to assess the difference in overall mortality between the elective and emergent cases.

The difference in mortality rate between ASA PS 5/5E was assessed with chi-square tests, and the difference in mortality rate at 48 hours and 30 days for all cases identified as ASA PS 5/5E was tested using an exact binomial test. Kaplan-Meier curves were estimated to test the independent effect of emergency classification on 30 and 90-day all-cause mortality in a cohort of patients classified as ASA PS 5/5E; data was censored at 30 or 90 days, as appropriate. A Mann-Whitney U-test was used to test the differences in LoS between ASA 5/5E. Finally, a Fisher’s exact test was used to assess the difference in the proportion of ASA PS 5 and 5E status levels in different discharge dispositions (in-hospital death, home, and other: hospice, long-term care facility, inpatient rehabilitation facility, or self-discharge). Patient characteristics and outcomes are presented descriptively (i.e., mean, SD, median, IQR, frequency, and percent).

All analyses were performed in Stata SE, Version 15 (College Station, TX), and significance was assessed at the 0.05 level. The Institutional Review Board at Weill Cornell Medicine approved all study activities (Protocol #180201900).

## Results

Sample characteristics

**Figure 1:**
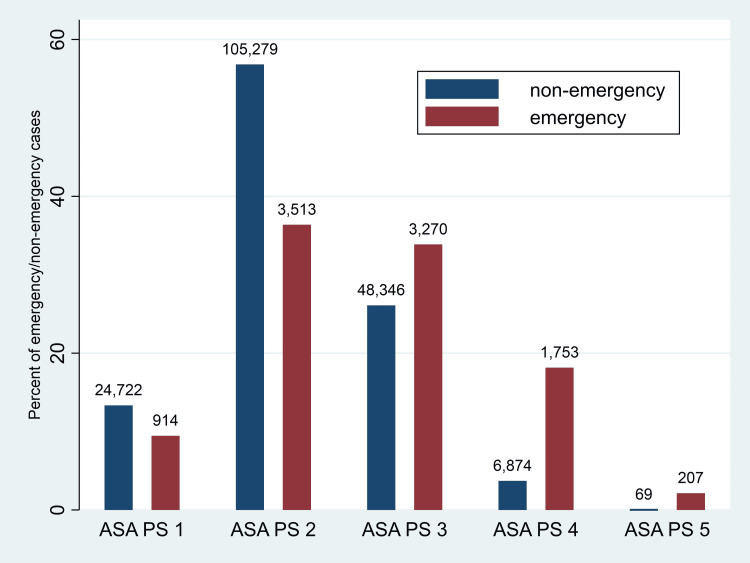
Distribution of ASA status by emergency status. ASA PS = American Society of Anesthesiologists Physical Status.

There were a total of 194,945 cases available for analysis. We excluded cases with missing values on age (N = 51); cases under 18 (N = 21,437); cases with ASA statuses of 6 (N = 19) or missing (N = 3); duplicates in terms of case identifier (N = 7); cases with a missing LoS (N = 13,462), and cases with a negative LoS (N = 12,205). Figure 1 displays the distribution of cases by ASA PS status and emergency classification in the final sample.

Overall, 5.0% of cases (N = 9,657) were identified as emergent (“E”). Table 1 contains patient demographic and preoperative measures. Two hundred seventy-four cases were identified as ASA PS 5 or 5E. Of those cases, 67 (24.4%) were classified as ASA PS 5, and 207 (75.6%) were classified as ASA PS 5E. The average age (years) for ASA PS5 was 59.2 ± 16.3 and for ASA PS 5E was 60.6 ± 18.0. 37.3% of the SA PS 5 sample was female, as was 36.7% of the ASA PS 5E sample. The most common procedures in the ASA PS 5 sample were cardiac/aortic (21.5%), integumentary (20.0%) and GI-endoscopy (15.4%) In the ASA PS 5E group, the most common surgeries were abdominal (29.5%), cardiac/aortic (24.6%), and neurologic (22.2%). There were no significant differences between age, sex, estimated blood loss, red blood cell transfusion; highest and lowest invasive MAP readings, lowest non-invasive MAP reading, and high and lowest temperature readings bet the two groups. 

**Table 1:** Patient demographics and preoperative measures, by emergency status. P-values represent a comparison between non-emergency and emergency status populations. Numbers reported are N (percentage) or median (interquartile range), as appropriate. SD = standard deviation; MAC = monitored anesthesia care; CSE = combined spinal-epidural; GI = gastrointestinal; MSK = musculoskeletal; OB-GYN = obstetrics/gynecology; ICU = intensive care unit; PACU = post-anesthesia care unit; EBL = estimated blood loss; RBC = red blood cells; MAP = mean arterial pressure; HR = heart rate.

	ASA PS-5 (N=67)	ASA-PS 5E (N=207)	Total (N=274)	P-value
Age (mean, SD)	59.16 (16.33)	60.57 (17.97)	60.22 (17.57)	0.5713
Sex				0.930
Male	42 (62.7%)	131 (63.3%)	173 (63.1%)	
Female	25 (37.3%)	76 (36.7%)	101 (36.9%)	
Anesthesia type				0.004
General	54 (80.6%)	195 (94.2%)	249 (90.1%)	
MAC	12 (17.9%)	11 (5.3%)	23 (8.4%)	
CSE	1 (1.5%)	0 (0%)	1 (0.4%)	
General + Epidural	0 (0%)	1 (0.5%)	1 (0.4%)	
Surgical type				< 0.001
ABD	5 (7.7%)	61 (29.5%)	66 (24.5%)	
Airway	5 (7.7%)	3 (1.5%)	8 (2.9%)	
Cardiac/aortic	14 (21.5%)	51 (24.6%)	65 (23.9%)	
GI-Endoscopy	10 (15.4%)	8 (3.9%)	18 (6.6%)	
Integumentary	13 (20.0%)	2 (1.0%)	15 (5.5%)	
Interventional radiology	5 (7.7%)	3 (1.5%)	8 (2.9%)	
MSK	1 (1.5%)	1 (0.5%)	2 (0.7%)	
Neuro	4 (6.2%)	46 (22.2%)	50 (18.4%)	
OB-GYN	0 (0.0%)	1 (0.5%)	2 (0.7%)	
Thoracic	1 (1.5%)	16 (7.7%)	17 (6.3%)	
Urology	3 (4.6%)	2 (1.0%)	5 (1.8%)	
Vascular	4 (6.2%)	13 (6.3%)	17 (6.3%)	
Anesthesia start time				< 0.001
00:00 – 05:59	1 (1.5%)	28 (13.5%)	29 (10.6%)	
06:00 – 11:59	26 (38.8%)	43 (20.8%)	69 (25.2%)	
12:00 – 17:59	33 (49.3%)	69 (33.3%)	102 (37.2%)	
18:00 – 23:59	7 (10.5%)	67 (32.4%)	74 (27.0%)	
Initial postoperative destination				0.049
ICU	50 (75.8%)	166 (80.2%)	216 (79.1%)	
Intraoperative death	1 (1.5%)	15 (7.3%)	16 (5.9%)	
PACU	15 (22.7%)	26 (12.6%)	41 (15.0%)	
EBL (mL)	200 (25-500)	300 (100-1000)	300 (50-800)	0.0846
RBC Transfusion (mL)	700 (350-1400)	1050 (700-1925)	1050 (700-1750)	0.1031
Highest invasive MAP (mm Hg)	243 (129-300)	251 (173.5-291.5)	249.5 (165-292)	0.4153
Lowest invasive MAP (mm Hg)	44.5 (16-62)	41 (9-59)	43 (14-59)	0.5316
Highest noninvasive MAP (mm Hg)	86 (79.5-97)	108 (91-125)	104 (86-123)	0.0003
Lowest noninvasive MAP (mm Hg)	66 (53-74)	60 (45-75)	63.5 (46-75)	0.3389
Highest HR	114.5 (99-128)	143 (114-201)	133 (111-190)	< 0.001
Lowest HR	61 (30-75)	35 (0-57)	39 (23-62)	< 0.001
Highest temperature (^o^C)	36 (35-37)	36 (35-37)	36 (35-37)	0.2773
Lowest temperature (^o^C)	34 (30-36)	34 (32-35)	34 (31-35)	0.3563

Mortality

In the 48-hour postoperative period, 146 patients (0.1%) out of 194,945 expired. The majority of patients (63.3%) who expired within 48 hours postoperatively independent of ASA classification were classified as “E.” By 30 days postoperatively (including those within 48 hours), a total of 1,187 (0.6%) patients out of 194,945 had expired, 34.2% of which were classified as “E.” There was an upward trend in the proportion of patients who expired postoperatively as ASA PS level increased (p < 0.01; data not shown); this same trend was also observed in subpopulations of emergent and non-emergent cases (both p < 0.01).

Mortality in ASA PS 5/5E

Mortality data for this population by emergency status and time period is shown in Table 2. Up to 48 hours, a larger percentage of ASA PS 5E patients died (15.5%, N = 32) than ASA PS 5 (6.0%, N = 4); however, this was not significant (p = 0.05). Between 49 hours and 30 days postoperatively, a larger percentage of ASA PS 5 patients died (34.9%, N = 22) than ASA PS 5E (23.0%, N = 40); this difference was not significant (p = 0.06). Between 31 and 90 days, a significantly larger percentage of the surviving ASA PS 5 population died (23.4%, N = 10) than the 5E population (7.4%, N = 10, p < 0.01).

**Table 2:** Mortality data for ASA 5 population, by emergency status. Death (non-cumulative, N, %). P-values represent a comparison between non-emergency and emergency status populations at each interval. ASA PS = American Society of Anesthesiologists Physical Status.

	0-48 hrs		49hrs – 30 days		31-90 days	
	At Risk	Death	At Risk	Death	At Risk	Death
ASA 5	69	5 (7.3%)	64	22 (34.4%)	42	10 (23.81%)
ASA 5E	207	32 (15.5%)	175	40 (22.6%)	135	10 (7.41%)
All ASA 5/5E	276	37 (13.4%) (p=0.08)	239	62 (25.9%) (p=0.07)	177	20 (11.30%) (p<0.01)

**Figure 2:**
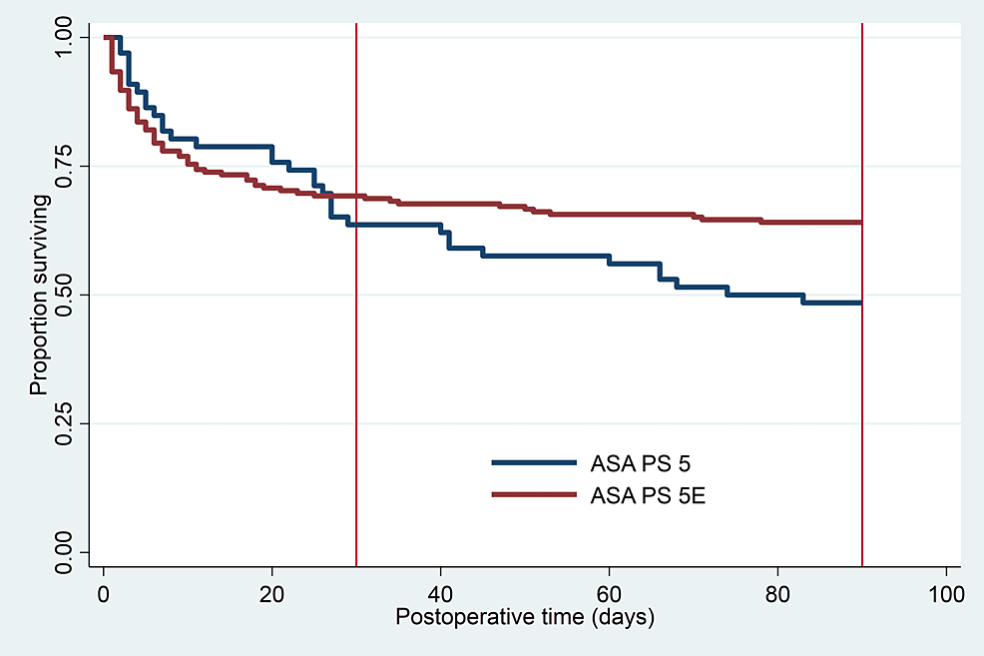
Kaplan-Meier survival curves for ASA PS 5 at 30 and 90 days postoperatively, by emergency status. Vertical lines identify 30- and 90-day time points. Y-axis represents the proportion of each population surviving, by day. ASA PS = American Society of Anesthesiologists Physical Status.

Overall, ASA PS 5/5E patients had a significantly higher mortality rate at 30 days than at 48 hours (25.1% vs. 13.1%, respectively, p < 0.01). However, a log-rank test for equality of the survivor functions at 30 and 90 days did not find a significant difference between 5 and 5E classifications (p = 0.58, p = 0.07, respectively; Figure 2.).

Length of stay (LoS)

Of the 62 ASA PS 5 patients who survived beyond 48 hours but expired before postoperative day 30, 40 (64.5%) had an emergency designation. Of the 177 ASA PS 5 patients surviving beyond 30 days postoperatively, 135 (76.3%) had an emergency designation. In neither population was the median LoS significantly different between elective and emergent groups (Table 3).

**Table 3:** Length of stay (LoS) in days, by emergency status. P-values represent comparison between non-emergency and emergency status populations at each interval. LoS = Length of stay; IQR = interquartile range. ASA PS = American Society of Anesthesiologists Physical Status.

		49 hours - 30 days (p=0.08)	N	31+ days (p=0.79)	N
LoS (median, IQR)	ASA PS 5	9.5 (5-25)	22	16 (4-43)	42
	ASA PS 5E	6.5 (4-11)	40	14 (7-34)	135
	All ASA PS 5s	7 (4-17)	62	15 (7-36)	177

Discharge disposition

**Figure 3:**
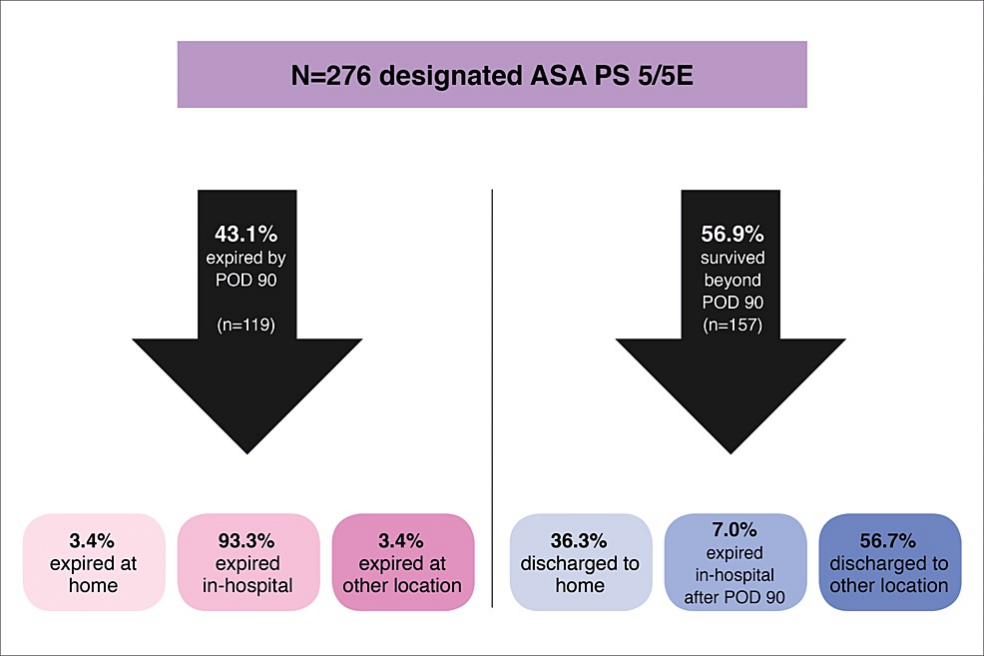
Discharge disposition of all ASA PS 5/5E patients. POD = postoperative day; ASA PS = American Society of Anesthesiologists Physical Status.

One hundred fifty seven of 274 (56.9%) patients survived beyond 90 days postoperatively, of which 125 (79.6%) had the emergency designation. Table 4 shows the discharge disposition data for patients who survived 0-48 hours, 49 hours-30 days, and 31-90 days postoperatively. There was no significant difference between ASA PS 5 and 5E in discharge dispositions of patients surviving beyond 90 days (p = 0.18). Of those patients surviving beyond 90 days postoperatively, the majority had been discharged to locations outside of the hospital, including hospice, long-term care facilities, inpatient rehabilitation, or self-discharged (Figure 3). 

**Table 4:** Discharge disposition of patients surviving by emergency status. Data represent the number of patients alive in the time period indicated. ASA PS = American Society of Anesthesiologists Physical Status.

Discharge disposition		Up to 48 hours	Up to 30 days	Up to 90 days
Home	ASA PS 5	13	13	12
	ASA PS 5E	48	47	45
In-hospital death	ASA PS 5	29	7	0
	ASA PS 5E	57	18	11
Other	ASA PS 5	22	22	20
	ASA PS 5E	70	70	69

## Discussion

Our study confirms the relationship between ASA PS classification and patient outcomes previously reported. We found an increasing mortality rate for patients with an increasing ASA PS classification score. To the best of our knowledge, our study is the first and only study to shed light on the postoperative destinations and LoS for surviving patients who were classified as moribund at the time of surgery and to discuss the implications of end-of-life saving surgery. This information should enable us to better inform our patients and their families about the likely postoperative outcomes and not just mortality, thereby improving their decision-making capacity.

Our analysis is based on physician assessment at the time of surgery. The ASA physical status classification system was introduced in 1941, and those patients with “extreme systemic disorders which have already become an imminent threat to life regardless of the type of treatment” were defined as Class 4 [14]. These individuals were subsequently denoted as Physical Status 5 (“moribund patients who are not expected to survive without the operation”) in the 1962 revision of the ASA classification system (and last amended on October 23, 2019), and the prefix (now suffix) “E” added to signify those patients “undergoing emergency procedures” [15]. Within this system, “Emergency” is defined as “existing when a delay in treatment of the patient would lead to a significant increase in the threat to life or body part” [16]. Then, as of now, the overall assessment of physical status is at the discretion of the anesthesiologist, leading to persistent, widespread, significant, inter-observer variability [6,17-21]. Even when clinicians are provided examples of the different physical status classes, variability persists, albeit at a lower rate [4]. Our analysis, in conjunction with the studies documenting the entrenched limits of the current classification system, suggests that a reassessment of the ASA physical status guidelines is in order, particularly for the most seriously ill patients.

Our results indicate that most patients who expired within the first 30 days postoperatively, irrespective of emergency status, did so in-hospital. The question thus arises: what is an ASA PS 5 patient that is not considered an emergency? Given the definitions provided in the ASA guidelines, it is illogical that a patient can be classified as ASA 5 but not 5E. We believe our data illustrates this point, as 30- and 90-day outcomes are similar in terms of mortality and ultimate patient disposition. Providing patients and their families a more complete picture of likely outcomes outside of the immediate perioperative period (i.e., <48 hours) is imperative in order for them to make an accurate and well-informed decision about potential end-of-life care.

Our data demonstrate that of patients classified as ASA PS 5 or 5E, 35.9% will expire in 30 days or less, and 98% of them will do so in hospital. In the context of this high mortality rate, is it ethical to take these patients to surgery or suggest that surgery will have a significant impact on the patient’s longevity without providing highly relevant information? With that, it is important to note that the majority of ASA PS 5 do in fact survive longer than 48 hours. This may have meaningful implications and impacts on end-of-life decision-making processes, considering patient values concerning goals of care and quality of life.

Greater than half of our patient population was alive at postoperative day 90, and unfortunately, most had not been discharged home but rather to a long-term hospital facility, sub-acute rehab facility, or hospice. It is imperative to highlight long-term patient outcomes when consenting patients for end-of-life surgeries. This will empower patients and families to make truly informed decisions not only on mortality but on quality of life.

Limitations

Our study has several limitations. One limitation is the inability to obtain all of the surgical indications for risk stratification, in addition to patient comorbidities. Similarly, this is a single-center study and may not be generalizable to the public at large and may be considered a small sample size in the age of large database research. Further, we could not control any individual incentives to up-score ASA or emergency status despite financial incentives being independent of patient or case type. Similarly, in a retrospective study, we were unable to control the operative surgeon and with such a short retrospective study there were no identifiable changes in surgical or hospital practices. Additionally, our database was unable to determine functional outcomes (i.e., living at home independently and/or disability-free survival). Finally, we could not independently verify ASA determination. The next step is to collaborate with other large academic programs to test our findings and address the generalizability of our dataset.

## Conclusions

In this single-center study, outcomes for moribund patients (classified as ASA PS 5) were similar to those designated as an emergency and non-emergency operative cases at all postoperative time points. Our analysis, in conjunction with the studies documenting the entrenched limits of the current physical status classification system, suggests that a reassessment of the ASA physical status guidelines is in order, particularly for the most seriously ill patients. Future research should delineate differences in outcomes using data from multiple institutions.
